# Comparison of craniofacial morphology in individuals with and without hypodontia with a special focus on the number of congenitally missing teeth

**DOI:** 10.3389/fpubh.2022.1013862

**Published:** 2022-11-18

**Authors:** Anita Fekonja, Andrej Čretnik

**Affiliations:** ^1^Department of Orthodontics, Community Healthcare Centre Maribor, Maribor, Slovenia; ^2^Faculty of Medicine, University of Maribor, Maribor, Slovenia

**Keywords:** hypodontia, craniofacial morphology, lateral cephalography, esthetics, congenital anomalies

## Abstract

**Background:**

Hypodontia might negatively affect dental function and esthetics, what might affect patients' self-esteem, communication behavior, professional performance and thus quality of life. The aim of this study was to estimate the influence of number of congenital missing teeth on dentofacial features.

**Methods:**

Lateral cephalograms of 60 individuals with hypodontia (study group) and 40 individuals without hypodontia (control group) were analyzed. Patients in the study group were divided into two subgroups according to the number of missing teeth (group A with hypodontia 1 to 4 teeth and group B with hypodontia 5 or more teeth). Cephalometric data were compared among the study and control groups and statistically analyzed.

**Results:**

The results in the present study revealed a significantly shorter and more retrognathic maxilla, more prognathic chin position, more retruded incisors in both jaws, large interincisal angle, straighter facial convexity as well as more retruded upper and lower lips in the group B compared with the control group. In the group A only chin position was significantly more prognathic compared with the control group.

**Conclusions:**

According to the results of present study impact of hypodontia on the craniofacial morphology and consequently on facial esthetics was found statistically significantly greater in patients with 5 or more congenitally missing teeth.

## Introduction

Congenitally missing teeth (CMT) or dental agenesis or hypodontia is one of the most common dental developmental anomalies, characterized by the absence of one or more deciduous or permanent teeth (1–3). CMT is defined when no mineralization of the tooth crown could be recognized on the radiograms and when the dental treatment records and anamnestic data expressed no extraction or loss because of trauma or caries (4). When a primary tooth is congenitally absent, its permanent counterpart might also be missing (3, 5). The prevalence of hypodontia in the permanent dentition, excluding the third molars, ranges between 0.15 and 16.2% and is depending on gender, race, and continent (1, 3, 4, 6, 7). The congenital absence of teeth occurs more frequently in girls at a ratio of 3:2. The mandibular second premolar and the maxillary lateral incisor are the most commonly missing teeth (2–4, 6). It has been reported that the occurrence of hypodontia in Caucasians has increased during the 20th century (7). Genetics plays a crucial role in congenital dental aplasia, as confirmed by studies on monozygotic twins (3, 8, 9). Its etiology seems to be multifactorial and can include environmental factors, infection, and drugs, as well as genes associated with about 120 syndromes (3). Hypodontia may be present as an isolated condition (10) or may be associated with other dental anomalies (1, 3, 11) and craniofacial syndromes such as cleft lip and/or palate, ectodermal dysplasia, and Down syndrome (3, 10, 12, 13). The isolated condition is more common than syndromic and can be sporadic or familiar (3).

Oral health plays an important role in public health, and hypodontia might negatively affect a patient's dental function and esthetic. Subjects with missing teeth are more likely to suffer from malocclusion, insufficient alveolar bone growth, masticatory problems, reduced chewing ability, inarticulate pronunciation (speech), periodontal damage, and esthetic problems with changes in skeletal relationships and an unfavorable appearance, which might negatively affect patients' self-esteem, communication behavior, professional performance, and thus the quality of life and might need multidisciplinary treatment (3, 14).

Based on severity, hypodontia can be classified into mild (two or fewer missing teeth), moderate (three to five missing teeth), and severe (six or more missing teeth) forms, with no clear agreement on that (2, 3, 15, 16).

The effect of hypodontia on craniofacial morphology has been widely studied. Hypodontia might accompany reduced intercanine and intermolar widths (2, 17). The results pertaining to skeletal changes remain controversial. Some authors did not find a significant correlation between malocclusions and hypodontia prevalence while according to the others studies significant impact could be found (2, 3). Acharya et al. (18) found in their study clinical significance associated only with patients with severe hypodontia (six or more missing teeth). Johal et al. (16) also found significant reductions in the 3D soft tissue morphology in patients with severe hypodontia (six or more missing teeth). It was found that only 0.14% of individuals with hypodontia show an absence of more than six permanent teeth (6, 16).

The purpose of the present study was to estimate the possible impact of even less than six congenitally missing permanent teeth on craniofacial skeletal as well as soft tissue relationships in our population.

## Materials and methods

Ethical approval for the study was obtained from the Institutional Review Board of the University Clinical Centre Maribor (no. 19/11). The study was conducted in accordance with the Declaration of Helsinki at the Orthodontic Department of Community Healthcare Centre. Informed consent approval was obtained from each subject.

In this retrospective case-control study, two groups of subjects were included: the study group with a variable number of missing teeth (tooth agenesis) and the control group with all teeth other than the third molars present.

The study group included 60 Caucasian subjects (28 boys and 32 girls) with a mean age of 14.7 years (standard deviation [SD] ± 1.6 years). The study group subjects were further divided into two subgroups according to the number of missing teeth: In group A, there were subjects with hypodontia of one to four teeth, and in group B, subjects with hypodontia of five or more teeth.

After determining the study group, the age- and sex-matched control group was randomly selected. The control group consisted of 40 Caucasian subjects (18 boys and 22 girls) with a mean age of 15.2 years (standard deviation [SD] ± 2.9 years) with complete permanent dentition (excluding third molars), a Class I molar relationship, lack of crossbite, enough space in both arches (Little's irregularity index was in the range of 1–3 mm in which each contact was <1 mm), positive overbite, and overjet <4 mm, without noticeable asymmetry and with consistent facial proportions.

A congenitally missing tooth was identified as the one when no mineralization of the tooth crown could be recognized on the radiograms and when the dental treatment records and anamnestic data confirmed that it had not been extracted or lost because of trauma or caries. Subjects who were younger than 12 years were not included in the study due to the timeline of tooth development (3). Subjects with syndromes, developmental anomalies (alveolar cleft and/or palate), mandibular fracture, or deformity were also excluded from the study because of the possibility of severe irregularities in craniofacial development. Pre-orthodontic treatment panoramic radiogram and lateral cephalogram availability with no previous orthodontic treatment were mandatory inclusion criteria for all the subjects in both groups.

All lateral cephalograms (LC) were obtained with the same equipment (Planmeca Promax, Finland) by an experienced dental radiology engineer using a standardized technique. Subjects were properly shielded, standing, with the ear rods in the external auditory meatus, the teeth in the maximal intercuspation (centric occlusion), relaxed lips, and Frankfort's horizontal plane running parallel to the floor. The distance from the midsagittal plane of the subject's head and to the source of rays on the one side and the film on the other side was the same for each subject.

Morphological craniofacial features were assessed from the digital LC with Planmeca Romexis cephalometric software program by a single operator (AF).

The assessment for each patient was carried out twice at different times to prevent errors and was corrected after the mean parameters were calculated.

Twenty-two skeletal and 11 soft tissue landmarks were recorded for each cephalogram, and 25 skeletal and 14 soft tissue measurements were obtained for the study. Definitions of the used landmarks and angular and linear measurements are described in Tables 1–3 (19, 20). The linear and angular measurements were taken to the nearest of 0.1 mm and 0.5 degrees, respectively.

**Table 1:** Definition of the skeletal and soft tissue cephalometric landmarks.

**Abbreviation**	**Description**	**Definition**
**Skeletal landmarks**		
G	Glabella	The most prominent point of the anterior contour of the frontal bone at the level of the superior orbital ridges in the midsagittal plane
S	Sella	The geometric center of the sella turcica, the center of the pituitary fossa of the sphenoid bone
N	Nasion	The intersection of the internasal and frontonasal sutures, in the midsagittal planes
A	A point - subspinale	The deepest midline point on the contour of the maxillary alveolar process, between the anterior nasal spine and the prosthion
B	B point - supramentale	The deepest point in the concavity along the anterior border of the symphysis, between the infradentale and pogonion
Ans	Anterior nasal spine	The tip of the anterior nasal spine at the inferior margin of the piriform aperture, in the midsagittal plane
Pns	Posterior nasal spine	The tip of the posterior nasal spine, the most posterior point on the contour of the bony hard palate in the midsagittal plane
Ar	Articulare	A constructed point representing the intersection of the inferior surface of the cranial base and the posterior outlines of the condylar head
Me	Menton	The lowest point of the contour of the mandibular symphysis, in the midsagittal plane
Gn	Gnathion	The most inferior point on the bony chin (mandibular symphysis) in the midsagittal plane
Pg	Pogonion	The most anterior point on the contour of the bony chin, in the midsagittal plane
Co	Condylion	The most superior and posterior point on the head of the mandibular condyle
Go	Gonion	The most posterior inferior point on the outline of the angle of the mandible
tGo	Tangent gonion	The intersection between the mandibular plane (mp) and the ramus plane (rp)
Is	Incision superius	The incisal point of the most prominent upper central incisor
Ii	Incision inferius	The incisal point of the most prominent mandibular central incisor
As	Apex superius	The root apex of the most prominent upper incisor
Ai	Apex inferius	The root apex of the most prominent lower incisor
Pr	Prosthion	The most inferior anterior point on the maxillary alveolar process, between the central incisors in the midsagittal plane
Id	Infradentale	The most superior anterior point of the mandibular alveolar process, between the central incisors in the midsagittal plane
Po	Porion	The most superior point of the outline of the external auditory meatus
Or	Orbitale	The lowest point on the inferior orbital margin
		Soft tissue landmarks
G'	Soft tissue glabella	The most prominent point of the soft tissue drape of the forehead, in the midsagittal plane
N'	Soft tissue nasion	The deepest point of the concavity between the forehead and the soft tissue contour of the nose in the midsagittal plane
Pn'	Pronasale	The most prominent or anterior point of the nose tip, in the midsagittal plane
Cm'	Columella	Nasal septum tangent point
Sn'	Subnasale	The point in the midsagittal plane where the base of the columella of the nose meets the upper lip
Ls'	Labrale superior	The most prominent point located on the vermilion border of the upper lip, in the midsagittal plane
Li'	Labrale inferior	The most prominent point located on the vermilion border of the lower lip, in the midsagittal plane
St'	Stomion	The most anterior point of the contact between the upper and lower lips in the midsagittal plane
Pg'	Soft tissue pogonion	The most prominent or anterior point on the soft tissue contour of the chin, in the midsagittal plane
Me'	Soft tissue menton	The most inferior point of the soft tissue chin, in the midsagittal plane
Sls, A'	Superior labial sulcus, soft tissue A	The deepest point in the concavity on the contour of the upper lip between subnasale and labrale superius, in the midsagittal plane

**Table 2:** The angular and linear skeletal measurements and their description.

**Measurements**	**Definition**
**Angular measurements**	
SNA	Prognathism of the maxillary alveolar bone, angle formed between the SN plane and point A
SNB	Prognathism of mandibular alveolar bone, angle formed between the SN plane and point B
ANB	Difference between SNA and SNB angles
SN/Pg	Prognathism of the mandible, angle formed between SN plane and point Pg
FH/NA	Angle formed between the Frankfort plane (Po-Or) and point A
FH/NPg	Facial angle, angle formed between the Frankfort plane (Po-Or) and point Pg
Go angle	Angle between the posterior tangent line of the ramus and the mandibular plane
SN/PP	Inclination of the maxilla, angle between the SN plane and the palatal plane
SN/MP	Inclination of the mandible, angle between the SN plane and the mandibular plane
Interbasal angle	Interbasal relationship, angle between the palatal (Ans–Pns) and mandibular (line tangent to the lower mandibular border) planes
U1/PP	Maxillary incisor inclination, angle between the upper central incisor (Is–As) and the palatal plane
L1/MP	Mandibular incisor inclination, angle between the lower central incisor (Ii–Ai) and the mandible plane
U1/L1	interincisal angle, angle between Is–As and Ii–Ai
**Linear measurements**	
S–N	Anterior cranial base length, distance between S and N
Ans–Pns	Length of the maxilla along the nasal floor, distance between ANS
Go–Pg	Length of the mandibular body, distance between Go and Pg at MP
Co–Pg	Length of mandible, distance between Co and Pg
PFH	Posterior face height (PFH), distance between S and Go
AFH	Anterior face height (AFH), distance between N and Me
PFH/AFH	Ratio between posterior and anterior face heights
UAFH	Upper anterior face height (UAFH), distance between N and Ans
LAFH	Lower anterior face height (LAFH), distance between Ans and Me
Wit's appraisal	Jaws anteroposterior relation to the cranial reference planes; projecting points A and B in two perpendicular lines, along the functional occlusal plane to get points AO and BO. Distance between points AO and BO; the plane is Wit's appraisal
Overjet	Horizontal distance from the upper incisor tip to the labial surface of the lower incisor
Overbite	Vertical distance from the lower incisor tip to the upper incisor tip

**Table 3:** The angular and linear soft tissue measurements and their descriptions.

**Measurements**	**Definition**
**Angular measurements**	
TFC	Total facial convex, angle, angle from G' to Pn' and to Pg'
FC	Facial convex angle, angle from G' to Sn' and to Pg'
NL angle	Nasolabial angle, angle between columella, Sn' and Sls (A')
**Linear measurements**	
TFL	Total facial length, distance between G' and Me'
FH	Facial height, distance between N' and Me'
LFH	Lower facial height, distance between Sn and Me
ULL	Upper lip length, distance between Sn' and St'
LLL	Lower lip length, distance between St' and Me'
UL thickness	Upper lip thickness, distance between Ls and labial surface of upper incisor
LL thickness	Lower lip thickness, distance between Li and labial surface of lower incisor
Ls/E	Upper lip convexity, distance of Ls to E line (Pn'–Pg')
Li/E	Lower lip convexity, distance of Li to E line (Pn'–Pg')
Pg–Pg'	Distance between pogonion and soft tissue pogonion
Me–Me'	Distance between menton and soft tissue menton

### Statistical analysis

The data were analyzed using the Statistical Package for Social Sciences, version 10.0 (SPSS Inc., Chicago, Illinois, USA). One examiner performed all measurements.

Fifteen randomly selected cephalograms from both groups were re-digitizing 2 weeks after initial digitization to evaluate intra-operator error. A paired *t*-test was used to perform error analysis. The difference was not statistically significant.

An independent *t*-test was used to compare cephalometric measurements between groups. The level of significance tested was *p* < 0.05.

## Results

Intra-operator error analysis showed no statistically significant difference observed in the cephalometric analyses between groups (*p* > 0.05). The correlation values were free of systematic measurement error (correlation coefficients were over 0.790).

There was no statistically significant difference between the patients in the study and control groups in age (*p* = 0.872) (Table 4).

**Table 4:** Distribution of subjects by groups.

	**Control group**	**Study group**	
		**A (1 to 4 missing teeth)**	**B (5 or more missing teeth)**
Patients (n)	40	40	20
Age (years; mean ± SD)	15.20 ± 2.9	14.95 ± 1.8	14.36 ± 1.4
Sex (female/male)	22/18	21/19	11/9

In the study groups, there were a total of 186 congenitally missing teeth [mean 1.78, SD 0.86; range 1–4 (for group A) and mean 5.75, SD 1.55; range 5–11 (for group B)].

Table 5 shows the distribution of missing teeth in both arches by the study group A and group B. The maxillary lateral incisor (39.5 %) was the most commonly absent tooth (affected with hypodontia) in group A while in group B the most commonly absent teeth were mandibular second premolar (30.4 %), followed by the maxillary lateral incisor (27.8 %) and maxillary second premolar (22.6 %).

**Table 5:** Distribution of type of missing teeth in upper and lower arches in groups with hypodontia.

**Groups** **Tooth type**	**Group A** **N (%)**	**Group B** **N (%)**	**p**
Upper lateral incisor	28 (39.5 %)	32 (27.8 %)	0.137
Upper first premolar	0 (0 %)	2 (1.8 %)	
Upper second premolar	14 (19.7 %)	26 (22.6 %)	0.777
Upper first molar	0 (0 %)	3 (2.6 %)	
Upper second molar	0 (0 %)	3 (2.6 %)	
Lower central incisor	2 (2.8 %)	5 (4.3 %)	0.891
Lower lateral incisor	0 (0 %)	2 (1.8 %)	
Lower canine	2 (2.8 %)	1 (0.9 %)	0.670
Lower second premolar	24 (33.8 %)	35 (30.4 %)	0.750
Lower first molar	0 (0 %)	2 (1.8%)	
Lower second molar	1 (1.4 %)	4 (3.4 %)	0.702
Total	71 (100 %)	115 (100 %)	

The data for all the cephalometric measurements of hard tissues and soft tissues with statistical analysis are presented in Tables 6, 7, respectively.

**Table 6:** Comparison of the skeletal cephalometric means and standard deviation (SD) between the control and study groups.

**Measurements**	**Control group**	**Study group**		**Control group vs. group A**	**Control group vs. group B**
		**A (1 to 4 missing teeth)**	**B (5 or more missing teeth)**		
**Angular skeletal and dentoalveolar dimensions (°)**					
SNA	80.83 ± 3.23	79.20 ± 4.61	76.17 ± 2.36	0.871	<0.001
SNB	78.27 ± 2.10	79.55 ± 5.69	76.83 ± 5.14	0.168	0.217
ANB	2.51 ± 1.90	1.31 ± 2.57	0.66 ± 3.63	0.055	0.023
SNPg	80.14 ± 3.21	83.05 ± 5.71	85.17 ± 3.27	0.003	<0.001
FH/NA	88.11 ± 3.05	87.20 ± 5.36	84.85 ± 2.97	0.388	<0.001
FH/NPg	86.67 ± 3.36	86.88 ± 6.46	86.52 ± 3.56	0.783	0.881
Go angle	121.70 ± 5.64	122.89 ± 6.29	120.67 ± 7.99	0.425	0.576
SN/PP	8.84 ± 3.07	7.47 ± 3.25	7.83 ± 2.23	0.085	0.217
SN/MP	32.65 ± 5.37	30.67 ± 6.22	31.50 ± 5.75	0.303	0.710
Interbasal angle	23.65 ± 5.59	22.68 ± 5.16	20.83 ± 9.11	0.513	0.183
U1/PP	109.63 ± 7.67	111.19 ± 8.75	94.67 ± 9.23	0.464	<0.001
L1/MP	90.59 ± 6.39	89.67 ± 6.02	85.67 ± 5.05	0.503	0.024
U1/L1	133.02 ± 8.80	135.09 ± 7.85	150.67 ± 4.22	0.353	<0.001
**Linear skeletal and dentoalveolar dimension (mm)**					
S–N	68.44 ± 3.32	69.07 ± 3.54	69.05 ± 2.76	0.911	0.932
Ans–Pns	52.03 ± 3.15	51.92 ± 3.03	49.8 ± 2.53	0.895	0.046
Go–Pg	72.47 ± 3.58	73.62 ± 5.69	72.50 ± 3.45	0.183	0.624
Co–Pg	107.71 ± 5.68	111.54 ± 8.84	110.85 ± 5.23	0.060	0.277
PFH	73.71 ± 5.11	73.33 ± 6.28	74.40 ± 2.51	0.796	0.622
AFH	112.48 ± 7.83	112.85 ± 7.33	116.75 ± 5.73	0.857	0.087
PFH/AFH	66.33 ± 4.94	67.19 ± 4.95	68.50 ± 6.25	0.479	0.171
UAFH	49.48 ± 3.39	51.02 ± 3.92	50.33 ± 3.07	0.062	0.370
LAFH	61.56 ± 4.21	62.25 ± 4.73	61.60 ± 3.94	0.649	0.978
Wit's appraisal	−1.25 ± 2.06	−1.85 ± 3.59	−0.67 ± 3.05	0.435	0.428
Overbite	3.19 ± 1.50	3.05 ± 1.28	3.83 ± 1.88	0.610	0.497
Overjet	3.05 ± 1.81	2.78 ± 1.66	2.17 ± 1.47	0.508	0.430

**Table 7:** Comparison of the soft tissue cephalometric means and standard deviation (SD) between the control and study groups.

**Measurements**	**Control group**	**Study group**		**Control group vs. group A**	**Control group vs. group B**
		**A (1 to 4 missing teeth)**	**B (5 or more missing teeth)**		
**Angular dimensions (** **°** **)**					
TFC	137.89 ± 4.94	139.71 ± 5.48	138.83 ± 5.74	0.245	0.675
FC	160.69 ± 5.97	162.86 ± 6.93	166.67 ± 6.40	0.263	0.034
NL angle	107.23 ± 10.82	109.86 ± 7.05	105.33 ± 10.17	0.258	0.318
**Linear dimensions (mm)**					
TFL	125.26 ± 5.90	127.64 ± 6.44	124.67 ± 9.37	0.204	0.791
FH	117.42 ± 6.27	120.07 ± 7.80	116.33 ± 8.71	0.059	0.627
LFH	65.06 ± 4.20	66.50 ± 5.26	62.17 ± 6.65	0.220	0.092
ULL	20.23 ± 2.37	20.64 ± 2.68	20.33 ± 2.66	0.496	0.494
LLL	44.74 ± 2.37	45.43 ± 3.11	43.50 ± 3.23	0.452	0.211
UL thickness	11.34 ± 1.53	11.63 ± 1.41	10.66 ± 1.97	0.283	0.162
LL thickness	10.54 ± 1.17	10.33 ± 1.17	9.67 ± 1.51	0.236	0.020
Ls/E	−4.15 ± 1.76	−4.77 ± 2.45	−6.83 ± 2.22	0.227	<0.001
Li/E	−2.53 ± 2.04	−3.14 ± 2.36	−5.40 ± 1.58	0.244	0.004
Pg–Pg'	10.78 ± 1.43	10.07 ± 1.97	10.16 ± 1.47	0.103	0.140
Me–Me'	7.17 ± 1.38	6.50 ± 1.60	6.67 ± 1.21	0.076	0.185

### Skeletal variables

There was one statistically significant difference noted in the measurements between study group A (one to four missing teeth) and the control group [SNPg variable (*p*= 0.003)] and eight out of 25 measurements for study group B (five or more missing teeth) in comparison with the control group: sagittal skeletal reduction with reduced SNA (*p* < 0.001), FH/NA (*p* < 0.001) and ANB angles (*p* = 0.023), shorter maxilla (p= 0.046), larger SNPg angle (*p* < 0.001), retrusion of upper (*p* < 0.001) and lower incisors (*p* = 0.024), and with increased interincisal angle (*p* < 0.001).

### Soft tissue variables

In the results of the present study, there were no statistically significant differences noted in soft tissue measurements between the patients in study group A and the control group.

On the other hand, the patients in study group B, (five or more missing teeth) statistically significantly straighter facial convexity (*p* = 0.034), retrusion of the upper (*p* < 0.001) and lower lips (*p* = 0.004), and smaller thickness of the lower lip (*p* = 0.020) were noted in comparison with the patients in the control group.

## Discussion

Hypodontia is a common developmental dental anomaly that might affect the growth pattern of the maxilla and mandible and consequently craniofacial morphology (2, 3).

In the present study, the most commonly missing tooth in group A was the maxillary lateral incisor, which is in agreement with the study by Endo et al. (21) who reported that anterior tooth agenesis was predominant in subjects with one or two missing teeth. The most commonly missing teeth in group B was the mandibular second premolar, followed by the maxillary lateral incisor and the maxillary second premolar, which is consistent with the results of previous studies (1, 4, 6, 7).

Ben-Bassat and Brin (22), Endo et al. (23), Nodal et al. (24), and Gungor and Turkkahraman (25) found that as the number of missing teeth increases, the influence of hypodontia on craniofacial morphology increases as well. Acharya et al. (18) found in their study clinically significant changes only in patients with six or more missing teeth. These findings were not in correlation with those of some other studies in which hypodontia was found to have little or no effect on craniofacial morphology (26, 27).

As in our population, there are not many subjects with six or more missing teeth (severe hypodontia), the primary intention of our study was to analyze whether even five or fewer missing teeth could have a significant impact on cephalometric parameters in our population. Indeed, the results of the present study revealed some statistically significant differences in skeletal and soft tissue measurements between the study and control groups in our population.

Cranial base measurements were similar in both (study and control) groups, while Endo et al. (23) and Woodworth et al. (28) evaluated a significantly shorter cranial base. The results of the present study revealed a significantly shorter maxilla (*p* = 0.046) in group B (hypodontia of five or more teeth) which correlates with those of some other studies (22, 23, 28, 29). During the eruption of the teeth, the alveolar processes of the maxilla and mandible grow rapidly, while teeth act as a functional matrix (30). Some authors reported that a shorter maxilla might be related to an anterior reduced development of the alveolar process because of the absence of the anterior teeth (25, 31–33) or by an insufficient apposition to the tuberosity area in posterior tooth agenesis (23). Besides the shorter maxilla, we observed also statistically significantly more retrognathic maxilla (SNA; *p* < 0.001 and FH/NA; *p* < 0.001) in group B (hypodontia of 5 or more teeth), which is similar to the studies that reported the smaller SNA angle (18, 22, 23) in subjects with severe hypodontia. Chan et al. (34) reported significantly reduced SNA and NAFH values in the severe hypodontia subgroup compared with the mild (hypodontia of one or two teeth) and moderate hypodontia subgroups (hypodontia of three to five teeth). Miševska et al. (29) reported a significantly reduced maxillary length in all three hypodontia groups [mild (1–2), moderate (3-6), severe (>7 congenitally missing permanent teeth)] compared with the controls. On the other hand, Roald et al. (26), Yuksel and Ucem (27), and Chung et al. (35) reported that hypodontia had little or no effect on growth patterns and consequently on SNA angles.

In the present study, no significant effect of hypodontia on the mandible length was found. A significantly larger SNPg angle was found in both hypodontia groups compared with the control group. Similar findings of the more prognathic mandible were published by Endo et al. (21, 23), Nodal et al. (23), Yuksel and Ucem (27), and Woodworth et al. (28), while Krezi et al. (31), Ben-Bassat and Brin (32), and Jurek et al. (36) on the contrary, reported retrognathic mandible.

The ANB was found to be significantly smaller in group B (*p* = 0.023), which is similar to the reports by Celikogu et al. (2), Acharya et al. (18), Miševka et al. (29), Chan et al. (34), and Chung et al. (35) who reported a greater tendency to a Class III skeletal relationship in severe hypodontia.

None of the vertical skeletal measurements in our study showed any statistically significant difference between the study and control groups. Subjects with hypodontia had no statistically significantly smaller SN/PP, SN/ML, and interbasal angles than the control group, and the effect of hypodontia increased only in subjects with five or more missing teeth, which is different from the results by Nodal et al. (24), Gungor and Tukkahraman (25), Ogaard and Krogstad (33), and Chan et al. (34) who showed significantly smaller mandibular plane inclination.

Comparison of dental relationships in our study revealed a statistically significant retrusion of the incisors in both jaws in study group B (hypodontia of five or more teeth) compared with the control group. Retrusion of the incisors might be a consequence of retroclination of the anterior teeth into the increased space caused by CMT. These results are similar to those of some other studies (21–23, 25, 29, 33, 37), while Yüksel and Ücem (27) reported a tendency toward protrusion of incisors in both jaws.

The human face undoubtedly plays a key contribution to an individual's perceived physical attractiveness, with people constantly being judged on their facial appearance (16, 33). In the results of our study, the interincisal angle was found statistically significantly greater (*p* < 0.001) in study group B than in the control group. This was accompanied by reduced lip protrusion. A significant retrusion of the incisors and an increased interincisal angle were observed with increasing severity of hypodontia by other authors, too (21, 23, 25, 29, 33). People who have well-positioned incisors might be considered more attractive than others who have dental malocclusion and/or missing teeth, which might be considered also as a psychological impact (11, 38).

The results obtained in the present study of face height showed no statistically significant differences between the study and control groups. On the other hand, in some previous studies (16, 25, 28, 29, 31, 33, 39), reports that subjects with hypodontia had a significantly shorter face compared with the control group can be found.

The findings of our study with no statistically significant differences in soft tissue measurements in the study group A (hypodontia of one to four teeth) in comparison with the control group are similar to the study by Roald et al. (26). However, in study group B, (hypodontia of five or more teeth) statistically significantly straighter facial profile, as well as more retrusive upper and lower lips, were found in comparison with the control group, which is in agreement with some other studies (29, 32, 33, 39).

Our study has some limitations, too. There were a relatively low number of subjects included in the study subgroup B (hypodontia of five or more teeth) due to the low number of our population. The limited sample size of this study in one location restricts validity to the larger population, but there was a statistically significant difference observed in eight out of 25 (32%) studied parameters, which is similar to the reports of other studies (31, 35). These craniofacial characteristics of subjects with hypodontia should be taken into consideration when treatment is planned, as reported by Endo et al. (21, 23), too.

Some authors (21, 23, 31) also studied the effects of the distribution of CMT on craniofacial morphology. The absence of one or two permanent teeth was the most common occurrence in our patients, which is consistent with some studies (2–4, 6). Although we have not found statistically significant differences in the distribution of CMT between groups, when studying the impact of hypodontia on craniofacial morphology apart from the number of CMT, it is necessary to take into account the distribution of CMT, too.

Another possible limitation of the study could be related to the subjects' age and the fact that both jaws might still grow during adolescence ages which should be taken into consideration regarding the average age of studied subjects in our as well as in other studies with the similar average age of subjects (26, 28, 34).

## Conclusion

In the results of the present study, we found that subjects affected by hypodontia indicate differences in craniofacial skeletal and soft tissue characteristics. Statistically significant differences were found in the group with five or more CMT.

The findings of the present study might suggest consideration of the changed craniofacial skeletal and soft tissue characteristics in subjects with hypodontia in a multidisciplinary approach, treatment planning, and care of possible functional and esthetic problems in such patients.

## Data availability statement

The original contributions presented in the study are included in the article/supplementary material, further inquiries can be directed to the corresponding author.

## Ethics statement

The studies involving human participants were reviewed and approved by Institutional Review Board of the University Clinical Centre Maribor (No. 19 / 11). Written informed consent to participate in this study was provided by the participants' legal guardian/next of kin.

## Author contributions

AF received the idea for the study, collected the data, and analyzed the data. Both authors contributed to the writing the article, in full review and approved the submitted version.

## Conflict of interest

The authors declare that the research was conducted in the absence of any commercial or financial relationships that could be construed as a potential conflict of interest.

## Publisher's note

All claims expressed in this article are solely those of the authors and do not necessarily represent those of their affiliated organizations, or those of the publisher, the editors and the reviewers. Any product that may be evaluated in this article, or claim that may be made by its manufacturer, is not guaranteed or endorsed by the publisher.

## References

[1] Larmour CJ Mossey PA Thind BS Forgie AH Stirrups DR. Hypodontia–a retrospective review of prevalence and etiology. Part I Quintessence Int. (2005) 36:263–70. 15835422

[2] Celikoglu M Kazanci F Miloglu O Oztek O Kamak H Ceylan I. Frequency and characteristics of tooth agenesis among an orthodontic patient population. Med Oral Patol Oral Cir Bucal. (2010) 15:797–801. 10.4317/medoral.15.e797 20383097

[3] Rakhshan V. Congenitally missing teeth (hypodontia): a review of the literature concerning the etiology, prevalence, risk factors, patterns and treatment. Dent Res J. (2015) 12:1–13. 10.4103/1735-3327.150286 25709668 PMC4336964

[4] Fekonja A Fekonja A. Hypodontia prevalence over four decades in a slovenian population. J Esth Rest Dent. (2013) 27:37–43. 10.1111/jerd.12076 24341573

[5] Hall RK. Congenitally missing teeth — A diagnostic feature in many syndromes of the head and neck. J Int Assoc Dent Child. (1983) 14:69–75. 6586857

[6] Polder BJ. Van't Hof MA, Van der Linden FPGM, Kuijpers-Jagtman AM. A meta-analysis of the prevalence of dental agenesis of permanent teeth. Comm Dent Oral Epidemiol. (2004) 32:217–26. 10.1111/j.1600-0528.2004.00158.x 15151692

[7] Mattheeuws N Dermaut L Martens G. Has hypodontia increased in Caucasians during the 20th century? A meta-analysis. Eur J Orthod. (2004) 26:99–103. 10.1093/ejo/26.1.99 14994889

[8] Militi D Militi A Cutrupi MC Portelli M Rigoli L Matarese G. Genetic basis of non syndromic hypodontia: a DNA investigation performed on three couples of monozygotic twins about PAX9 mutation. Eur J Paediatr Dent. (2011) 12:21–4. 21434731

[9] Arte S. Phenotypic and genotypic features of familial hypodontia. [dissertation]. [Helsinki (Finland)]: Institute of Dentistry, University of Helsinki, Finland. (2001). Available online at: http://ethesis.helsinki.fi/julkaisut/laa/hamma/vk/atre/phenotyp.pdf (accessed July 2, 2021).

[10] Dhamo B Kuijpers MAR Balk-Leurs I Boxum C Wolvius EB Ongkosuwito EM. Disturbance of dental developmental distinguish patients with oligodontia-ectodermal dysplasia from isolated oligodontia. Orthod Craniofac Res. (2018) 21:48–56. 10.1111/ocr.12214 29271123

[11] Fekonja A. Prevalence of dental developmental anomalies of permanent teeth in children and their influence on esthetic. J Esthet Restor Dent. (2017) 12302:1–8. doi. 10.1111/jerd.12302 28509361 10.1111/jerd.12302

[12] Slayton RL Williams L Murray JC Wheeler JJ Lidral AC Nishimura CJ. Genetic association studies of cleft lip and/or palate with hypodontia outside the cleft region. Cleft Palate Craniofac. (2003) 40:274–9. 10.1597/1545-1569_2003_040_0274_gasocl_2.0.co_2 12733956 PMC2752356

[13] Ghaib NH Al-Khatieeb MM Abd DH. Hypodontia in Down syndrome patients. J Bagh Coll Dentistry. (2009) 21:98–103.

[14] Pithon MM Vargas EOA da Silva Coqueiro R Lacerda-Santos R Tanaka OM Maia LC. Impact of oral-health-related quality of life and self-esteem on patients with missing maxillary lateral incisor after orthodontic space closure: a single-blinded, randomized, controlled trial. Eur J Orthod. (2021) 21:208–14. 10.1093/ejo/cjaa075 33367539

[15] Hobkirk JA Goodman JR Jones SP. Presenting complaints and findings in a group of patients attending a hypodontia clinic. Br Dent J. (1994) 177:337–9. 10.1038/sj.bdj.4808606 7980981

[16] Johal A Hasan E Zou LF Wong F Shahdad S Al-Klash R. The influence of mild versus severe hypodontia on facial soft tissue? A three-dimensional optical laser scanning-based cohort study. J Orthod. (2021) 48:33–41. 10.1177/1465312520967016 33118457

[17] Fekonja A. Comparison of mesiodistal crown dimension and arch width in subjects with and without hypodontia. J Esthet Restor Dent. (2013) 25:203–10. 10.1111/jerd.12026 23773516

[18] Acharya PN Jones SP Moles D Gill AD Hunt NP. A cephalometric study to investigate the skeletal relationships in patients with increasing severity of hypodontia. Angle Orthod. (2010) 80:511–8. 10.2319/072309-411.1 20482356 PMC8966445

[19] Proffit WR Sarver DM Fields HW Jr. Orthodontic Diagnosis: The Problem-Oriented Aprroach. In: Proffit WR Fields HW Larson BE Sarver DM, editirs. Contemporary orthodontics. 6th ed. Mosby, St Louis. (2018). p. 140-205.

[20] Drevenšek M Farčnik F Vidmar G. Cephalometric standards for Slovenians in the mixed dentition period. Eur J Ortod. (2006) 28:51–7. 10.1093/ejo/cji081 16230328

[21] Endo T Ozoe R Yoshino S Shimooka S. Hypodontia patterns and variations in craniofacial morphology in Japanese orthodontic patients. Angle Orthod. (2006) 76:996–1003. 10.2319/082905-303 17090161

[22] Ben-Bassat Y Brin I. Skeletal and dental patterns in patients with severe congenital absence of teeth. Am J Orthod Dentofacial Orthop. (2009) 135:349–56. 10.1016/j.ajodo.2008.09.002 19268834

[23] Endo T Yoshino S Ozoe R Kojima K Shimooka S. Association of advanced hypodontia and craniofacial morphology in Japanese orthodontic patients. Odontology. (2004) 92:48–53. 10.1007/s10266-004-0034-5 15490305

[24] Nodal M Kjaer I Solow B. Craniofacial morphology in patients with multiple congenitally missing permanent teeth. Eur J Orthod. (1994) 16:104–9. 10.1093/ejo/16.2.104 8005197

[25] Gungor AY Turkkahraman H. Effects of severity and location of nonsyndromic hypodontia on craniofacial morphology. Angle Orthod. (2013) 83:584–90. 10.2319/091012-722.1 23311600 PMC8754045

[26] Roald KL Wisth PJ Boe OE. Changes in cranio-facial morphology of individuals with hypodontia between the ages of 9 and 16. Acta Odontol Scand. (1982) 40:65–74. 10.3109/00016358209041117 6954829

[27] Yuksel S Ucem T. The effect of tooth agenesis on dentofacial structures. Eur J Orthod. (1997) 19:71–8. 10.1093/ejo/19.1.71 9071047

[28] Woodworth DA Sinclair PM Alexander RG. Bilateral congenital absence of maxillary lateral incisors: a craniofacial and dental cast analysis. Am J Orthod. (1985) 87:280–93. 10.1016/0002-9416(85)90003-X 3857005

[29] Miševska C Kanurkova L Bajraktarova Valjakova E Georgieva S Bajraktarova B Georgiev Z. Craniofacial morphology in individuals with increasing severity of hypodontia. South Eur J Orthod Dentofac Res. (2016) 3:12–7. 10.5937/sejodr3-126611204676

[30] Brodie AG. The growth of alveolar bone and the eruption of the teeth. Oral Surg Oral Med Oral Path. (1948) 1:342–5. 10.1016/0030-4220(48)90257-6 18910808

[31] Kreczi A Proff P Falermeier R Faltermeier C. Effect of hypodontia on craniofacial structures and mandibular growth pattern. Head Face Med. (2011) 7:23–31. 10.1186/1746-160X-7-23 22142280 PMC3248361

[32] Ben-Bassat Y Brin I. Skeletodental patterns in patients with multiple congenitally missing teeth. Am J Orthod Dentofacial Orthop. (2003) 124:521–5. 10.1016/S0889-5406(03)00620-6 14614419

[33] Ogaard B Krogstad O. Craniofacial structure and soft tissue profile in patients with severe hypodontia. Am J Orthod Dentofacial Orthop. (1995) 108:472–7. 10.1016/S0889-5406(95)70047-1 7484966

[34] Chan DWS Samman N McMillan AS. Craniofacial profile in Southern Chinese with hypodontia. Eur J Orthod. (2009) 31:300–5. 10.1093/ejo/cjn111 19193707

[35] Chung LK Hobson RS Nunn JH Gordon PH Carter NE. An analysis of the skeletal relationships in a group of young people with hypodontia. J Orthod. (2000) 27:315-8. 10.1093/ortho/27.4.315 11099569

[36] Jurek A Gozdowski D Czochrowska EM Zadurska M. Effect of tooth agenesis on mandibular morphology and position. Int J Environ Res Public Health. (2021) 18:11876–86. 10.3390/ijerph182211876 34831629 PMC8625843

[37] Sarnas KV Rune B. The facial profile in advanced hypodontia: a mixed longitudinal study of 141 children. Eur J Orthod. (1983) 5:133–43. 10.1093/ejo/5.2.133 6574919

[38] Beall AE. Can a new smile make you look more intelligent and successful? Dent Clin North Am. (2007) 51:289–97. 10.1016/j.cden.2007.02.002 17532913

[39] Bondarets N McDonald F Analysis of the vertical facial form in patients with severe hypodontia. Am J Phys Anthropol. (2000) 111:177–84. 10.1002/(SICI)1096-8644(200002)111:2<177::AID-AJPA4>3.0.CO;2-83.0.CO;2-8 10640945

